# Effect of inulin from dahlia tubers (*Dahlia variabilis*) extract on insulitis severity and insulin expression in diabetic rats

**DOI:** 10.37796/2211-8039.1460

**Published:** 2024-09-01

**Authors:** Ismawati, Saryono, Mukhyarjon, Ilhami Romus, Veni D. Putri, Sri Yanti, Fitri Dyna, Nada I. Adesti

**Affiliations:** a Faculty of Medicine, Universitas Riau, Pekanbaru, Indonesia; b Chemistry Study Program, Faculty of Mathematics and Natural Sciences, Universitas Riau, Pekanbaru, Indonesia; c Nursing Study Program, STIKes Payung Negeri Pekanbaru, Pekanbaru, Indonesia; d Nursing Profession Study Program, STIKes Payung Negeri Pekanbaru, Pekanbaru, Indonesia; e Medical Doctor Study Program, Faculty of Medicine, Riau University, Pekanbaru, Indonesia

**Keywords:** Insulin, Diabetes mellitus, Inulin, Dahlia tubers

## Abstract

**Background:**

Dahlia (*Dahlia variabilis*), a widely cultivated ornamental plant in Indonesia, is known to contain 84.08% inulin in its tubers. Numerous studies have demonstrated the antidiabetic potential of inulin from various plant sources. However, most of the research is in the form of a mixture of inulin with other active substances, and no one has analyzed the effects of inulin derived from dahlia tubers. This study examines the effect of inulin from dahlia tuber extract on blood glucose levels, serum insulin expression, pancreatic tissue insulin expression, homeostatic model assessment of insulin resistance (HOMA-IR), and the extent of insulitis in diabetic rats.

**Methods:**

In this experimental study, 20 male Wistar rats were randomly allocated to five groups. Group I served as the control, Group II as the STZ-induced diabetic group, Group III as the STZ-induced diabetic group treated with inulin (0.5 g/kgBW), Group IV as the STZ induced diabetic group treated with inulin (1.0 g/kgBW), and Group V as the STZ-induced diabetic group treated with inulin (1.5 g/kgBW). The inulin was administered for 21 days. The degree of insulitis was evaluated using a scoring system, serum insulin concentration via ELISA, and insulin expression in the pancreas through immunohistochemistry.

**Results:**

Administration of inulin from dahlia tubers significantly reduced serum glucose concentrations in diabetic rats. Notably, only inulin extracts at doses of 1 g/kgBW and 1.5 g/kgBW showed a significant reduction in insulitis and HOMA-IR index in diabetic rats, while the 0.5 g/kgBW inulin extract reduced insulitis without affecting HOMA-IR. Inulin extract administration did not affect insulin expression in serum or pancreatic tissue.

**Conclusions:**

Inulin from dahlia tuber can exert antidiabetic properties by improving insulin resistance and insulitis. These studies suggest the great potential of dahlia tubers as the source of inulin for prebiotic functional foods.

## 1. Introduction

Diabetes mellitus (DM), as defined by the American Diabetes Association, is a metabolic disorder characterized by hyperglycemia resulting from disruptions in insulin secretion or insulin function (insulin resistance), or both [1]. Currently, DM has emerged as one of the major global health concerns due to its significant contribution to morbidity in both developing and developed countries. The number of individuals affected by diabetes continues to increase annually. In 2021, approximately 537 million people worldwide will live with diabetes. Projections suggest that by 2030, the global diabetic population will reach 643 million (equivalent to 11.3% of the world’s population), and it is expected to rise to 783 million by 2045 [2].

Diabetes mellitus (DM) represents a health challenge. Over recent decades, lifestyles have shifted, particularly in adopting unhealthy dietary habits characterized by imbalanced nutrition, including high consumption of fats and sugars coupled with a fiber deficiency, all exacerbated by reduced physical activity. This trend has been linked to the escalating prevalence of obesity, a critical factor in the development of diabetes mellitus [3]. Conversely, several studies have demonstrated that a high-fiber, low-glucose, and low-transfat diet can protect against the onset of type 2 diabetes, emphasizing the critical role of diet in preventing and managing DM [4].

Inulin is a dietary fiber with potential as an antidiabetic agent [5]. Inulin is a polysaccharide composed of fructose molecules linked through β(2–1)d-fructofuranoside bonds. This bond structure makes inulin resistant to digestion by digestive enzymes, leading to delayed gastric emptying and slowing glucose absorption [6]. Research conducted by Nishimura et al. (2018) showed that chicory root extract containing inulin for four weeks in adults can reduce hemoglobin A1c (HbA1c) levels [7]. Another study in 2019 revealed that type 2 diabetes mellitus (T2DM) rats induced by a high-fat diet (HFD) and streptozotocin (STZ) for five weeks displayed significantly enhanced insulin sensitivity, increased glycogen synthesis, and improved glucose transport activation through the PI3K/Akt pathway [8]. Findings from research involving diabetic rats receiving *Lactobacillus plantarum* 1058 (ATCC 8014) and inulin supplements for eight weeks indicated reduced hyperglycemia, insulin resistance, hyper-lipidemia, oxidative stress, and increased insulin and leptin levels in the hypothalamus of the rats [9]. However, several clinical trial studies have shown controversial results [10]. Roshanravan’s research in 2017 showed that administration of 10 g/day of inulin supplementation singly could not improve fasting plasma glucose, insulin, HbA1C, and HOMA-IR in type 2 DM patients, but it is better when using inulin with butyrate supplement [11].

The previous studies have highlighted the potential of inulin derived from various plant sources as an effective antidiabetic agent. However, it is essential to note that the content of inulin varies significantly between different plants, both in terms of quantity and type. This variability is influenced not only by the plant species but also by the plant’s growth location, environmental conditions, and other factors. Even plants of the same species grown in different locations or environments can produce distinct bioactive compounds or compounds from the same group with varying levels of bioactivity [12].

Dahlia, a popular ornamental plant thriving in the highlands of Indonesia, offers a promising resource. Dahlia tubers, in particular, have the potential to serve as a functional food with the ability to prevent and treat diabetes due to their rich inulin content, which is a highly beneficial dietary fiber [5]. Despite this potential, the utilization of dahlia tubers in society remains suboptimal and is often regarded as agricultural waste [13].

In prior studies, the researchers extracted and characterized inulin from dahlia tubers. The findings confirmed that dahlia tubers contain 84.08% inulin. The production of inulin was achieved through an extraction process, and a proximate analysis of the inulin revealed its composition, including water content (6.75%), ash content (0.39%), crude protein (0.95%), fat (0.49%), carbohydrates (91.41%), and natural fiber (0.97%) [14,15]. Research on inulin from dahlia tubers is essential for advancing inulin as a functional food for individuals with type 2 diabetes. This study aims to assess the effects of administering inulin extracted from dahlia tubers (*Dahlia variabilis*) on blood glucose levels, insulin expression in both serum and pancreatic tissue, HOMA-IR, and the degree of insulitis in diabetic rats.

## 2. Materials and methods

### 2.1. Research design

This experimental research utilized a post-test with a Control Group Design. The study involved a total of 20 male Wistar rats randomly allocated into five groups, with each group consisting of four rats. Group I served as the control group, Group II as the STZ induced diabetic group, Group III as the STZ-induced diabetic group treated with inulin (0.5 g/kgBW), Group IV as the STZ-induced diabetic group treated with inulin (1.0 g/kgBW), and Group V as the STZ-induced diabetic group treated with inulin (1.5 g/kgBW) [16].

### 2.2. Inulin extraction

Inulin, which was the object of this study, was extracted from dahlia tubers (*Dahlia variabilis*). The dahlia tubers were sourced from fresh dahlia plants cultivated in Bukittinggi, West Sumatra, Indonesia. The extraction process began by heating 2500 g of cleaned and finely sliced dahlia tubers in 5000 ml of distilled water at temperatures ranging from 80 °C to 90 °C for 30 min. After cooling to room temperature, the mixture was filtered, resulting in a filtrate and residue. The filtrate was mixed with ethanol in a 1:1 (v/v) ratio and chilled at 4 °C for 18 h. Subsequently, the filtrate was centrifuged at 9000 rpm for 10 min, yielding a white inulin precipitate. This white precipitate was dried at 60 °C until it became a dry, off-white inulin powder.

### 2.3. Experimental animal handling

Healthy male Wistar rats with an average weight of 150–200 g were obtained from the STIFAR Riau Animal Research Laboratory (Indonesia). The rats were accommodated in a well-ventilated room at a temperature of 22 ± 2 °C, with an air humidity level of 60 ± 10%, and subjected to a 12-h light–dark cycle in the STIFAR Riau Animal Research Laboratory. Throughout the study, the animals were provided with standard diets and unrestricted access to water. Ethical considerations in treating the animals adhered to the established procedures of the Helsinki Convention. They were approved by the medical and health research ethics unit of the Faculty of Medicine, Riau University, under the ethical clearance number B/046/UN19.5.1.1.8/UEPKK/2023.

Before the treatment, the rats underwent a seven-day acclimatization period. The cages were cleaned daily, and wood shavings as bedding material were replaced every three days. The rats were subjected to a 12-h fasting period, after which they were intraperitoneally injected with a single dose of streptozotocin (STZ) at 60 mg/kg BW, prepared in a 0.1M citrate buffer at pH 4.5. Nicotinamide (NA) was administered at dose of 120 mg/kg BW 15 min later [16]. Blood samples were collected from the tail vein after 72 h. Fasting glucose levels were determined using the glucose oxidase phenol aminophenazone (GOD-PAP) method. A total of 10 μL of serum was mixed with 1000 μL of GOD-PAP reagent and vortexed. The solution was incubated at room temperature for 20 min and measured using a spectrophotometer. Rats with blood glucose levels exceeding 250 mg/dL were diagnosed with diabetes and included in the subsequent treatment with inulin. Inulin was administered to the rats daily via oral gavage using a feeding tube for 21 days [15].

### 2.4. Insulitis examination

Following anesthesia with ether, the pancreatic organs were retrieved and preserved in a 10% formalin buffer. The preparation of pancreatic tissue was conducted within the Pathological Anatomy Laboratory, and the samples were subjected to hematoxylin-eosin (HE) staining. The entire pancreatic tissue was adequately processed and assessed for Langerhans islets within ten fields of view. The evaluation was performed under a light microscope at a 400× magnification, employing a scoring system [17] as described: score 0 (normal) indicated the absence of lymphocytes within the Langerhans islets; score 1 (mild insulitis) signified the presence of some mononuclear inflammatory cells (lymphocytes) surrounding the Langerhans islets (periinsulitis); score 2 (moderate insulitis) indicated the presence of some mononuclear cells (lymphocytes) in a small portion of the Langerhans islets (<50%); and score 3 (severe insulitis) represented the presence of mononuclear inflammatory cells (lymphocytes) in the majority of the Langerhans islets (>50%) [18].

### 2.5. Serum insulin examination

Blood samples were collected from the heart, centrifuged at 3000 rpm to obtain serum, and stored at −80 °C until analysis. The serum insulin concentration was measured using an ELISA kit (ER1113, Wuhan Fine Biotech Co., Ltd, Wuhan, China).

### 2.6. Pancreatic insulin expression examination

The expression of insulin in the pancreatic tissue of Wistar rats was assessed using the immunohistochemical technique with a polyclonal insulin antibody (Fine Biotech Co., Ltd, Wuhan, China). The examination was performed under a light microscope with ten fields of view at 400× magnification. Insulin expression was categorized as follows: Negative (−) when no brown staining was observed, Weak Positive (+) when brown staining was present with low intensity, and Strong Positive (+) when brown staining was evident with high intensity [19].

### 2.7. Statistical analysis

The ANOVA test was employed to compare blood glucose levels statistically, insulitis scores in the Langerhans islets of the rat pancreas, serum insulin concentrations among the groups, HOMA-IR, and post hoc tests were conducted subsequently. A p-value of <0.05 was considered statistically significant.

## 3. Results

### 3.1. Experimental animal model

**Fig. 1:**
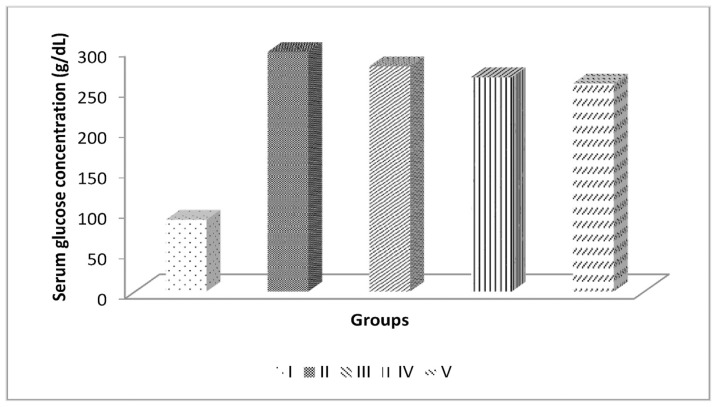
Fasting serum glucose concentration on the 3rd day of induction (mg/dL).

In experimental animal groups II and III, a random blood glucose test was performed three days after inducing diabetes with streptozotocin and nicotinamide. The average random blood glucose levels in groups II to V were higher than in group I. All the rats induced with diabetes had random blood glucose levels above 250 mg/dL. In contrast, the random blood glucose levels in the control group were below 250 mg/dL (Fig. 1).

### 3.2. Effect of inulin from dahlia tuber extract on fasting serum glucose concentration

**Fig. 2:**
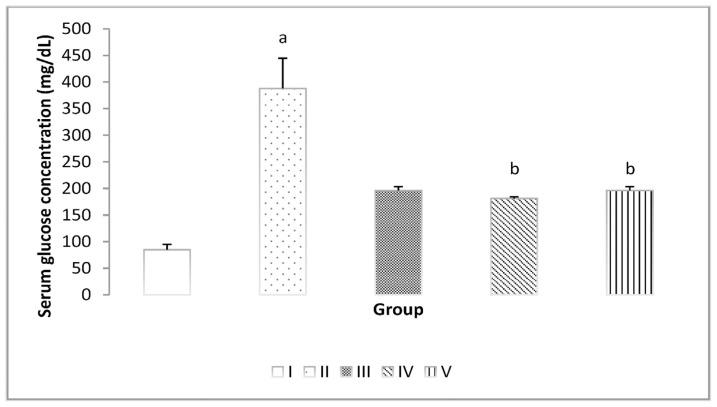
Serum glucose levels in the five groups after 21 days. Diabetes was induced by a single intraperitoneal injection of streptozotocin followed by intraperitoneal administration of nicotinamide. (a) P < 0.05 vs. control. (b) P < 0.05 vs. DM.

On day 22, the serum glucose examination results revealed a significant increase in serum glucose compared to the normal group. However, administering inulin from dahlia tuber extract at doses of 0.5, 1, and 1.5 g/kg significantly lowered the serum glucose concentration compared to Group II (DM group) (Fig. 2).

### 3.3. Effect of inulin from dahlia tuber extract on langerhans islet insulitis severity

**Fig. 3:**
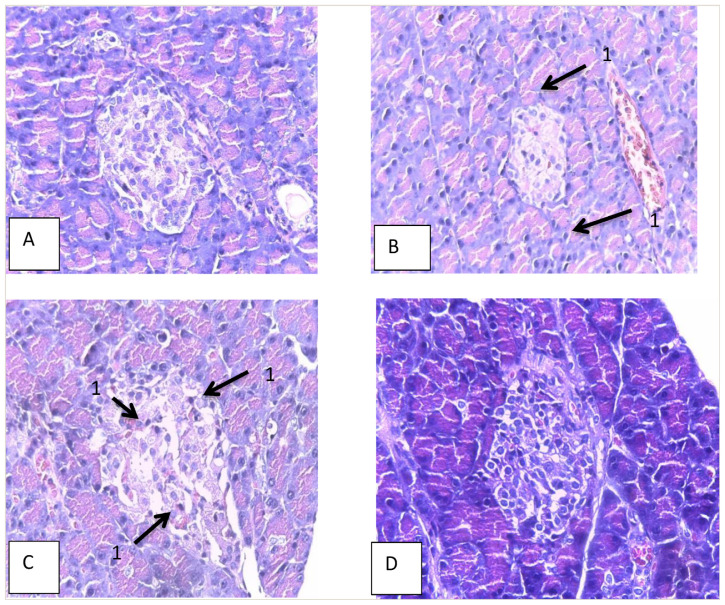
Histopathological Image of Rat Pancreatic Langerhans Islets in Each Treatment Group (HE Staining) (Magnification 400x); A = Group I; B = Group II, score 1; C = Group II, score 2; D = Group V, score 0; 1 = lymphocyte.

Table 1 and Fig. 3 display the results of microscopic examinations of the pancreas in different groups. The highest insulitis scores were observed in the Type 2 DM group (Group II), followed by the DM + inulin 0.5 g/kgBW group (Group III), the DM + inulin 1.5 g/kgBW group (Group V), and lastly, the control group (Group I). Group I (Fig. 3A), scoring 0 (normal), showed evenly distributed β cells with no lymphocyte infiltration or necrosis. In the Type 2 DM group (Fig. 3B) with a score of 1 (mild), lymphocytes were present around the Langerhans islets (peri insulitis). Group II, with a score of 2 (moderate) (Fig. 3C), exhibited lymphocytes within the Langerhans islets. The DM Type 2+inulin group (Fig. 3D), with a score of 0 (normal), showed no lymphocyte infiltration. The administration of inulin at doses of 0.5 g/kgBW, 1 g/kgBW, and 1.5 g/kgBW reduced insulitis in DM rats.

### 3.4. Effect of inulin from dahlia tuber extract on serum insulin concentration

**Fig. 4:**
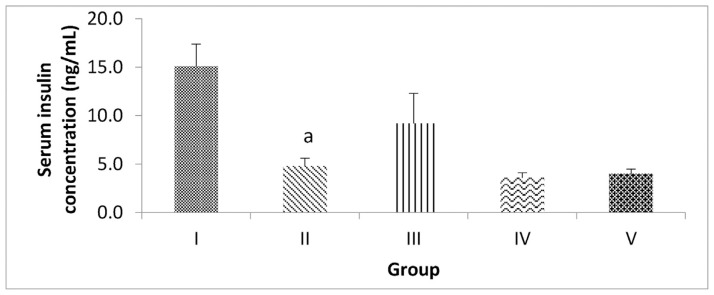
Serum insulin concentration in the five groups after 22 Days. Diabetes was induced by a single intraperitoneal injection of Streptozotocin, followed by intraperitoneal administration of Nicotinamide in Groups II–V. Inulin was administered in Groups III–V. After 21 days, serum insulin concentration was measured. (a) P < 0.05 vs. Control.

The serum insulin examination results (Fig. 4) show that the highest serum insulin concentration was in Group I (control), followed by Groups III, II, V, and IV. The data presented indicate a significant reduction in serum insulin concentration in DM-induced rat groups. The administration of inulin extract at a dose of 0.5 g/kgBW (Group III) increased serum insulin concentration, although not significantly.

### 3.5. Effect of inulin from dahlia tuber extract on the HOMA-IR index

**Fig. 5:**
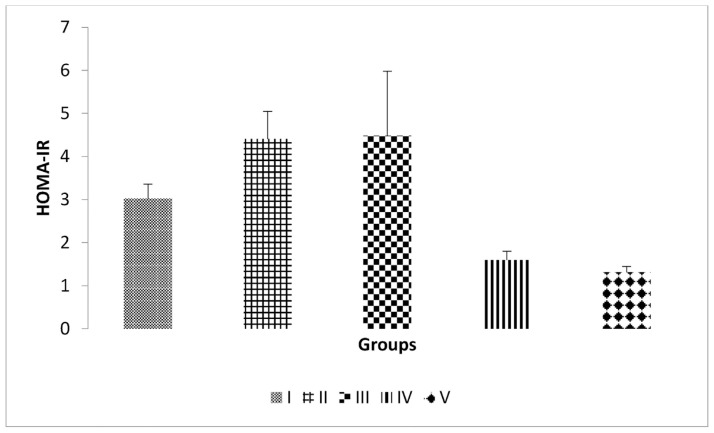
HOMA-IR in the five groups after 22 days. Diabetes was induced by a single intraperitoneal injection of Streptozotocin, followed by intraperitoneal administration of Nicotinamide in groups II–V. Inulin was administered in groups III–V. b: P < 0.05 vs. II.

The HOMA-IR index was calculated using the formula: fasting glucose level (mg/dL) x fasting insulin level (ng/mL)/405 [20] Fig. 5 illustrates that the mean HOMA-IR index in Group II (4.41) exceeded that in Group I (3.02). Administering inulin from dahlia tubers substantially reduced the HOMA-IR index in Groups IV and V compared to Group II.

### 3.6. Effect of inulin from dahlia tuber extract on insulin expression in pancreatic tissue

**Fig. 6:**
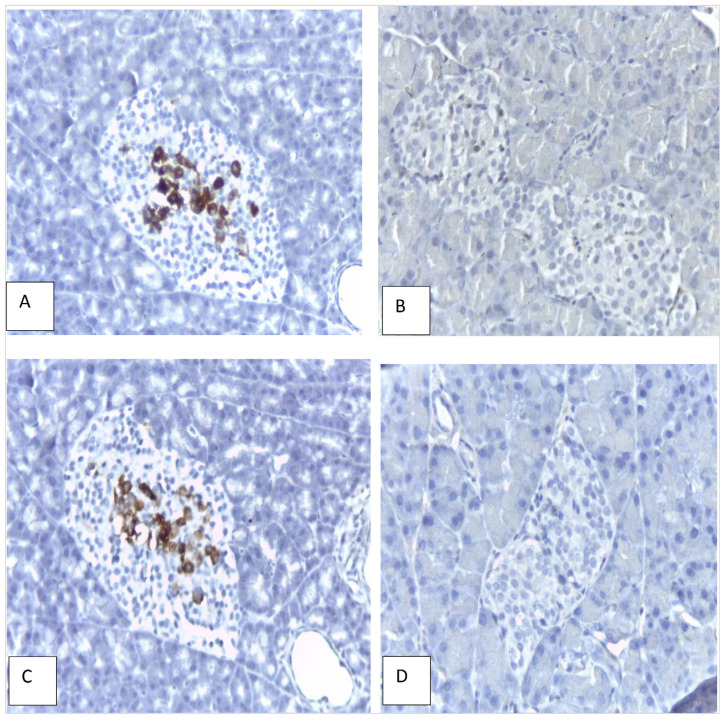
Immunohistochemical image of insulin expression in pancreatic tissue in the treatment groups (Magnification 400x). A: strong positive in Group I, negative in Group II, C: strong positive in Group III, negative in Group V. Diaminobenzidine stains the insulin brown. Slides were counterstained with H&E.

Table 2 illustrates insulin expression in all groups. In Group I, all rats displayed insulin expression, whereas in Group II, only 75% exhibited insulin expression with weak intensity. The administration of inulin from dahlia tubers did not enhance insulin expression in diabetic rats, although 25% displayed strong insulin expression in Group III (Fig. 6).

## 4. Discussion

In this study, the administration of STZ (60 mg/kgBW) followed by nicotinamide (120 mg/kgBW) given 15 min afterward significantly increased serum glucose concentration compared to the control group. STZ administration induces cell death by reducing nicotinamide adenine dinucleotide (NAD+) in pancreatic β-cells. Nicotinamide acts as a precursor to NAD+ and inhibits poly-ADP-ribose-polymerase-1 (PARP-1). NAD+ is a crucial coenzyme in ATP production and other metabolic pathways. Extensive DNA damage leads to the overactivation of PARP-1 and cell death. Nicotinamide administration protects pancreatic β-cells against the cytotoxic effects of STZ [21]. Intraperitoneal administration of STZ and nicotinamide induces type 2 diabetes in experimental animals [16]. Type 2 diabetes is characterized by elevated blood glucose levels, insulin resistance, and relative insulin deficiency [22].

In the present study, administration of inulin from dahlia tubers significantly reduced serum glucose concentrations and HOMA-IR index but did not substantially change insulin expression in serum or pancreatic tissue in diabetic rats. The outcome is consistent with earlier research regarding blood glucose levels. Several studies have explored the effect of inulin or inulin mixtures on glucose concentration, and the results generally suggest a positive association. This decrease provides further evidence of inulin’s hypoglycemic effects. Numerous studies have shown that the consumption of inulin can increase the bulkness, which lengthens the time it takes for your stomach to empty. Furthermore, they reduce the rate at which glucose is absorbed, which causes sugar to be released more slowly and can lower the postprandial insulin level. After leaving the upper gastrointestinal tract, a diet high in fiber does not change; instead, it ferments in the colon to produce short-chain fatty acids (SCFA), which alter insulin sensitivity and gluconeogenesis [6]. The hypoglycemic mechanism involves enhancing glucose transport through the insulindependent phosphatidylinositol 3-kinase/Akt (PI3-K/Akt) pathway [6,23].

Regarding the impact of inulin on insulin expression and HOMA-IR, conflicting results have been reported in earlier published studies. Zhang et al. conducted a meta-analysis to examine the efficiency of inulin in patients with T2DM. Analyses revealed a significant role of inulin supplementation in improving HOMA-IR and insulin expression when treatment duration eight we. In our study, 1 and 1.5 mg/kg BW of inulin extract can improve HOMA-IR, even if it did not increase insulin expression. It is consistent with results obtained from the administration of *Ganoderma lucidum* for five weeks in rats induced by a high-fat diet (HFD) and streptozotocin (STZ), which showed no increase in insulin secretion despite significant improvements in insulin sensitivity, substantial glycogen synthesis, and facilitated glucose transport through activation of the PI3K/Akt pathway [9]. This difference in the effects of inulin is likely due to differences in duration of administration and differences in physicochemical behavior. The physicochemical behavior of inulin is related to its daily intake and degree of polymerization (DP). The DP of different inulin sources is different and depends on processing history, growing conditions, weather, harvest time, and storage conditions [12]. Inulin may affect post-translational modifications (PTM) or activities of proteins involved in insulin signaling pathways without necessarily altering the overall expression of insulin. Accumulating evidence from recent studies suggests that SCFAs can mediate various PTM-dependent mechanisms, modulating protein activity and influencing cellular signaling [24].

In the current study, inulin administration significantly affected pancreatic tissue and alleviated insulitis. The pancreas, part of the gastrointestinal system, plays an essential role in the development of DM by producing the hormone insulin through the islets of Langerhans (β cells). Damage to pancreatic tissue, such as insulitis, can affect insulin production and the development of DM [25,26]. While insulitis has long been associated with the pathogenesis of type 1 diabetes, it is becoming evident through research that insulitis also occurs in type 2 diabetes. Despite differing causes, insulitis in type 1 and type 2 diabetes utilizes the same effector molecules and signal transduction pathways. The Böni-Schnetzler hypothesis proposes that hyperglycemia induces interleukin 1-β (IL-1 β) production. This, in turn, leads to increased IL-1 β production through auto-induction mechanisms and the production of other inflammatory factors, such as IL-8. Consequently, macrophages are activated to produce more IL-1 β, ultimately causing the death and dysfunction of pancreatic β-cells [27].

Inulin is known to modulate gut microbiota. Administration of fructan-type inulin for six weeks has enhanced gut microbiota in type 2 diabetes [28]. Various studies indicate that gut microbiota is crucial in inflammation and metabolic disorders such as obesity, insulin resistance, and type 2 diabetes. Multiple cytokines, such as tumor necrosis factor-alpha (TNF-a), interleukin-6 (IL-6), and high-sensitive C-reactive protein (hs-CRP), can trigger a set of inflammatory processes, insulin resistance, and dysfunction of beta cells [28,29]. The results of this study show that the administration of inulin at various concentrations can significantly reduce the degree of insulitis. These findings align with research on mice induced with diabetes using STZ and given an inulin-enriched diet for 12 weeks, demonstrating reduced inflammatory cytokine levels like MCP-1 and IL-6 in the pancreas. Interleukin 22 plays a potential role in the regeneration of Langerhans islet cells. Reg3 protein, stimulated by interleukin 22, is believed to act as a hormone that can prevent damage to pancreatic beta cells [30].

In conclusion, inulin from dahlia tuber can exert antidiabetic properties by improving insulin resistance and insulitis. Inulin from dahlia tuber can exert antidiabetic properties by improving insulin resistance and insulitis. These studies suggest the great potential of dahlia tubers as the source of inulin for prebiotic functional foods.

## Figures and Tables

**Table 1:** Mean Langerhans islet insulitis scores in rat pancreas in different groups.

Group	Mean ± SD	n
I	4	0,00
II	4	1.3 ± 0.1^a^
III	4	0.75 ± 0.2^b^
IV	4	0.56 ± 0.1^b^
V	4	0.6 ± 0.1^b^

* a = p < 0.05 compared to Group I,

* b = p < 0.05 compared to Group II, using ANOVA and post hoc tests.

**Table 2:** Insulin expression distribution in pancreatic tissue across various groups.

Insulin Expression	Group I (n = 4)	Group II (n = 4)	Group III (n = 4)	Group IV (n = 4)	Group V (n = 4)
Negative	0	1	3	3	4
Weak Positive	2	3	0	1	0
Strong Positive	2	0	1	0	0
